# Supplementary material for: Intrinsic topological magnons in arrays of magnetic dipoles
Source: Sci Rep. 2022 Jan 26;12:1420. doi: 10.1038/s41598-022-05469-4 (PMC8792029; doi:10.1038/s41598-022-05469-4)
Supplement: Supplementary file 1 — Supplementary Information 1. [file 41598_2022_5469_MOESM1_ESM.pdf]

Supplementary Information
Intrinsic topological magnons in arrays of magnetic dipoles

Paula Mellado[1,2]
[1]*School of Engineering and Sciences, Universidad Adolfo Ibáñez, Santiago, Chile*
[2]CIIBEC, Santiago, Chile.

A. Minimum energy configurations.

Figure 1 show screenshots obtained from the energy minimization of the total energy consisting of magnetic dipolar interactions among all dipoles and an easy axis anisotropy energy (Eq.1 in the paper) in arrays made out of 50 point dipoles. We consider the case of chains with $\kappa = \pi/2$ and hight anisotropy $K > K_c^\Lambda$ in (a) and (b), while in (c) we show the collinear state that result from minimizing chains at low anisotropy. (d) shows the case of a stripe at $K > K_c^\Lambda$ and $\Lambda \sim 1$. For small amplitudes $\Lambda \sim 0.1$, fig. 1(a) shows the antiferromagnetic order. For $\Lambda \sim 1$, figs. 1(b) and (d), a dimerized magnetic arrangement can be seen in these finite systems.

In Figure 2 we show chains with modulation of longer wavelenght. In cases with high anisotropy (a) and (b) show screenshots of chains with $\kappa = \pi/4$ and $\pi/8$ respectively. The collinear state at $K < K_c^\Lambda$ and $\kappa = \pi/8$ is shown in (c).

Figure 3 shows the total energy u, of modulated chains and stripes as a function of Λ at $K > K_c^\Lambda$. In all cases, at low Λ the magnetic configuration with the lowest energy consist in the antiferromagnetic state (AF). For intermediate Λ (~ 1) AF becomes energetically expensive and the two dimerized configurations 'up-up-down-down' and 'down-up-up-down' (*D1* and *D2*) are the lowest energy states. *D1* and *D2* are very close in energy.

B. Band spectrum of stripes for larger modulations.

Figure 4 shows results for the band spectrum of chains and stripes with modulation having larger wavelengths $\kappa = \pi/4$ and $\kappa = \pi/8$ at $K < K_c^\Lambda$ and $K > K_c^\Lambda$.

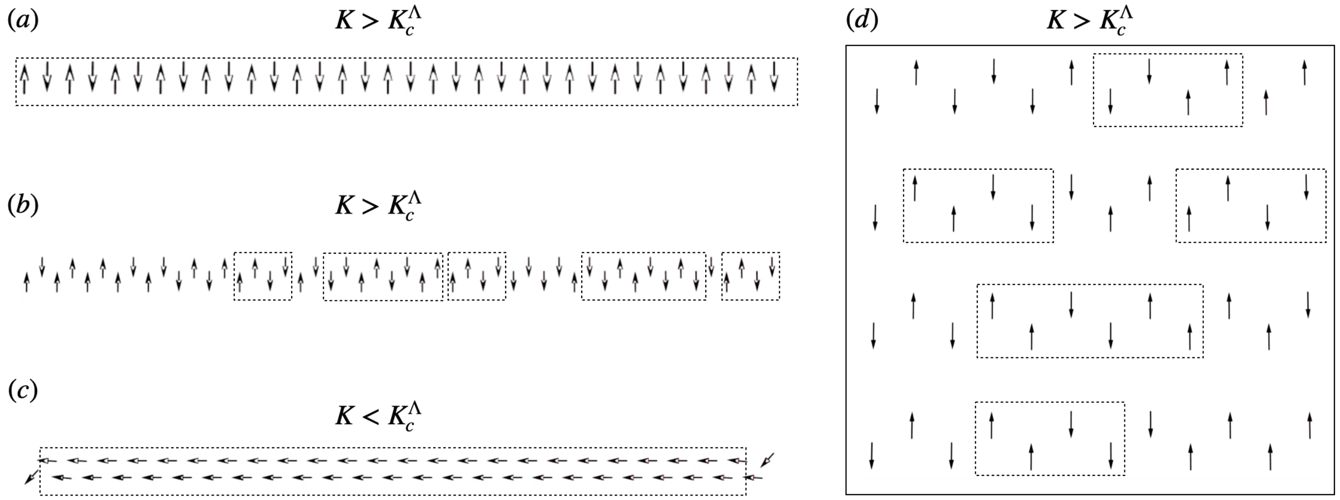

FIG. 1: Magnetic configurations that minimize the total energy Eq.1 in the paper in chains with $\kappa = \pi/2$ and (a) $K > K_c^\Lambda$, $\Lambda = 0.1$, (b) $K > K_c^\Lambda$, $\Lambda \sim 1$ and (c) $K < K_c^\Lambda$, $\Lambda \sim 1$. (d) stripe at $K > K_c^\Lambda$, $\Lambda \sim 1$.

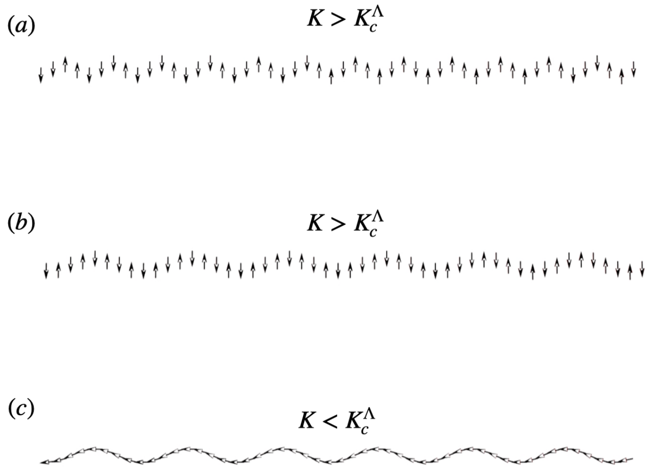

FIG. 2: Magnetic configurations that minimize the total energy Eq.1 in the following cases. (a) $K > K_c^\Lambda$ and $\kappa = \pi/4$, (b) $K > K_c^\Lambda$ and $\kappa = \pi/8$, (c) $K < K_c^\Lambda$ and $\kappa = \pi/8$ at $\Lambda \sim 1$.

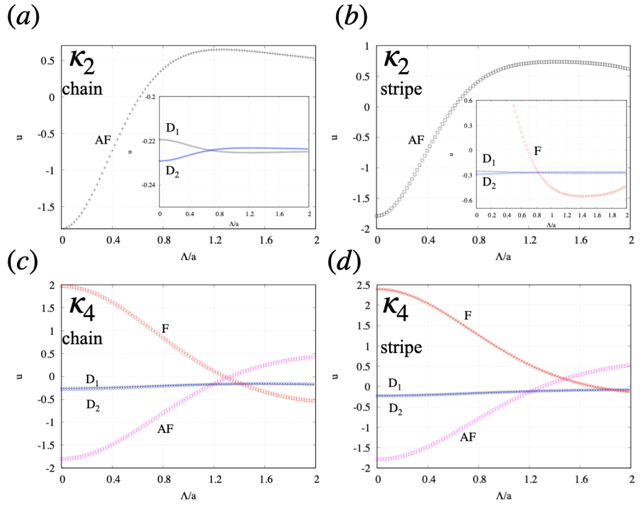

FIG. 3: Total energy u, of modulated chains and stripes as a function of Λ at $K > K_c^\Lambda$. (a) chain with $\kappa = \pi/2$, (b) stripe with $\kappa = \pi/2$, (c) chain with $\kappa = \pi/4$, (d) stripe with $\kappa = \pi/4$. The ferromagnetic configuration F is very expensive for the values of Λ used in this study.

C. Dipolar sums

The dipolar interaction reads,

$$\mathcal{H}^{dd} = \frac{\mathcal{D}}{2} \sum_{i \neq k=1}^{n} \frac{\hat{\boldsymbol{m}}_i \cdot \hat{\boldsymbol{m}}_k - 3(\hat{\boldsymbol{m}}_i \cdot \hat{\boldsymbol{e}}_{ik})(\hat{\boldsymbol{m}}_k \cdot \hat{\boldsymbol{e}}_{ik})}{|\boldsymbol{r}_i - \boldsymbol{r}_k|^3} \tag{1}$$

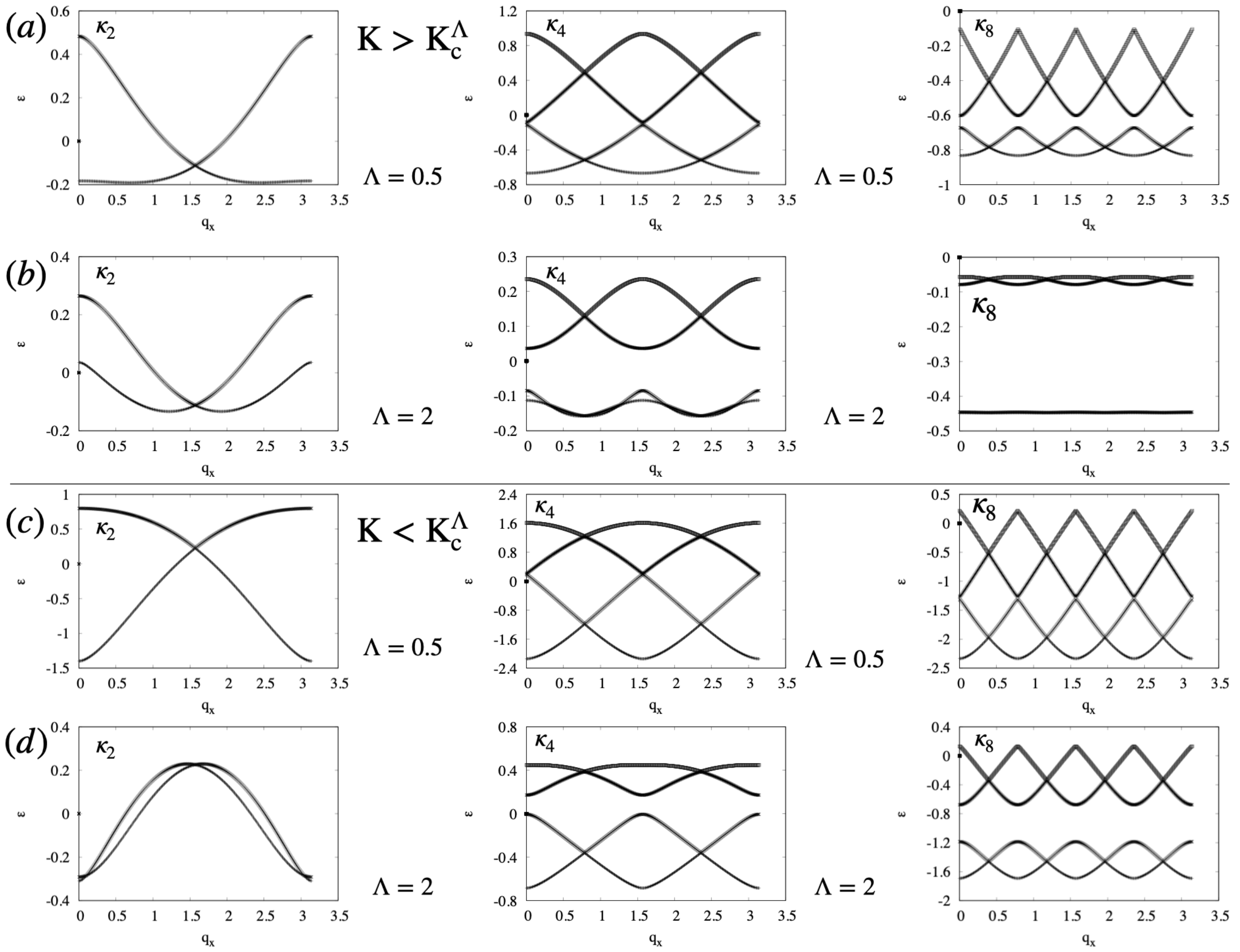

FIG. 4: Band spectrum of chains. (a) Column shows chains at $K > K_c^{\Lambda}$, with $\Lambda = 0.5$ and $\kappa = \pi/2$ (left panel), $\kappa = \pi/4$ (middle panel) and $\kappa = \pi/8$ (right). (b) same as in (a) but at $\Lambda = 2$. (c) same as in (a) but at $K < K_c^{\Lambda}$, (d) same as in (b) but at $K < K_c^{\Lambda}$.

1. *Dipolar sums for parallel magnets along $\hat{z}$.*

In the parallel ground state configuration (Section II in the paper) the dipolar sum between dipoles that belong to the same sublattice in the chain reads:

$$J_{q_x}^{(11)} = -\frac{1}{8}\sum_{n=1}^{\infty}\frac{\exp\left(2\imath qn\right)}{n^3} = -\frac{1}{8}s_1 = -\frac{1}{8}\mathrm{Li}_3\left(e^{2iq}\right) \tag{2}$$

Using that the polylogarithm can be approximated to $\mathrm{Li}_s\left(z\right) \sim \frac{2^s z}{2^s - z}$, we get

$$J_q^{(11)} \sim -\frac{1}{8\cos 2q - 1} \tag{3}$$

And the dipolar sum between dipoles that belong to different sublattices in the chain reads:

$$\mathrm{J}_{\mathrm{q}}^{(12)} = -\sum_{\mathrm{n}=0}^{\infty}\frac{\exp\left(\imath\mathrm{q}(2\mathrm{n}+1)\right)}{((2\mathrm{n}+1)^2+\Lambda^2)^{3/2}} + \Lambda^2\sum_{\mathrm{n}=0}^{\infty}\frac{\exp\left(\imath\mathrm{q}(2\mathrm{n}+1)\right)}{((2\mathrm{n}+1)^2+\Lambda^2)^{5/2}} = -\mathrm{s}_3 + \Lambda^2\mathrm{s}_4 \tag{4}$$

where
$s_1 = \sum_{n=1}^{\infty}\frac{\exp\left(2\imath qn\right)}{n^3} = \mathrm{Li}_3\left(e^{2iq}\right)$,

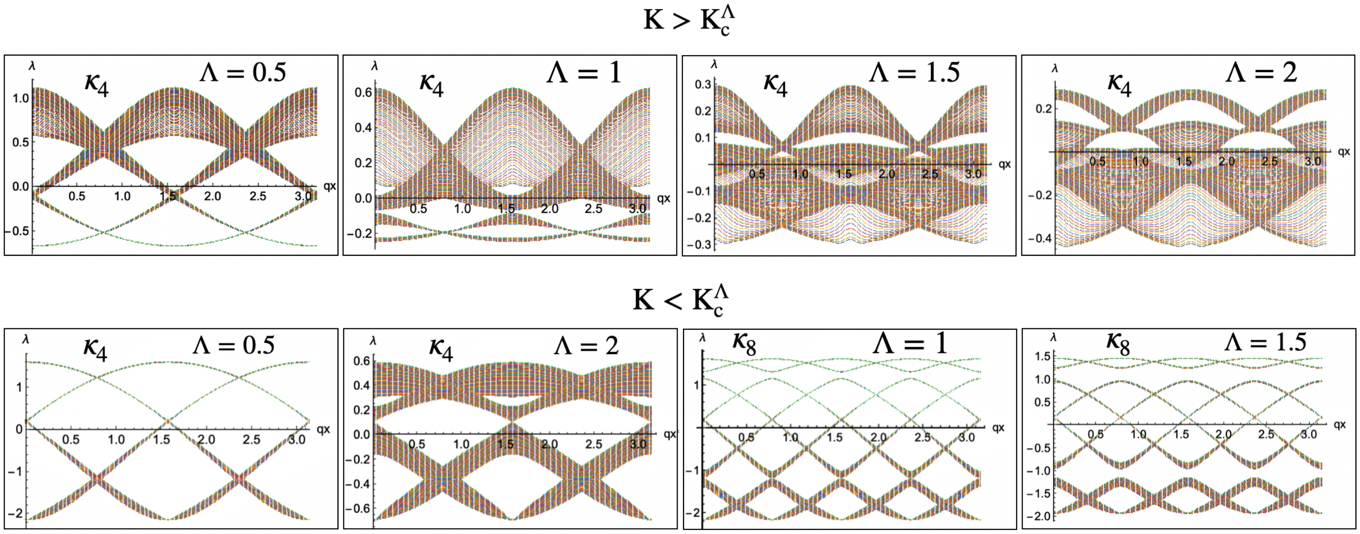

FIG. 5: Band spectrum of stripes with $\kappa = \pi/4$. Top panel: $K > K_c^\Lambda$. Lower panel: $K < K_c^\Lambda$.

$s_3 = \sum_{n=0}^{\infty} \frac{\exp(\imath q(2n+1))}{((2n+1)^2+\Lambda^2)^{3/2}} = e^{iq\sqrt{1+\Lambda^2}} \Phi\left(e^{iq}, 3, \sqrt{1+\Lambda^2}\right)$ and
$s_4 = \sum_{n=0}^{\infty} \frac{\exp(\imath q(2n+1))}{((2n+1)^2+\Lambda^2)^{5/2}} = e^{iq\sqrt{1+\Lambda^2}} \Phi\left(e^{iq}, 5, \sqrt{1+\Lambda^2}\right)$.

We can approximate $\Phi\left(e^{iq}, 3, \sqrt{1+\Lambda^2}\right) \sim \Phi\left(e^{iq}, 3, 1\right)$ and likewise $\Phi\left(e^{iq}, 5, \sqrt{1+\Lambda^2}\right) \sim \Phi\left(e^{iq}, 5, 1\right)$ and we get,

$$s_3 \sim \frac{8\cos(q\sqrt{1+\Lambda^2})}{(8-\cos(2q))} \tag{5}$$

$$s_4 \sim \frac{32\cos(q\sqrt{1+\Lambda^2})}{(32-\cos(2q))} \tag{6}$$

To give

$$\mathrm{J_q^{(1,2)}} \sim 8\cos(\mathrm{q}\sqrt{1+\Lambda^2})\left(-1+\frac{4\Lambda^2}{(32-\cos(2\mathrm{q}))}\right) \tag{7}$$

D. Critical anisotropy.

Depending on Λ and $\mathcal{K}$, finite modulated chains of point dipoles minimize (in the paper) Eq.1, in the magnetic configurations illustrated (in the paper) Fig.1. For small anisotropy $\mathcal{K} < \mathcal{K}_c^\Lambda$, the collinear magnetic order, Figs.1(c-d) is favored, while for $\mathcal{K} > \mathcal{K}_c^\Lambda$ the parallel magnetic states (Figs.1(a-b)) minimize energy. One can find the critical anisotropy by balancing the total energy of the system in a parallel magnetic configuration

$$\epsilon^{\text{parallel}} = \mathcal{D}(2J_z^{(11)} + J_z^{(12)}) - \sum_{n=1}^{\infty} \frac{\mathcal{K}}{2} \tag{8}$$

with the total energy in the case of a collinear magnetic configuration,

$$\epsilon^{\text{collinear}} = \mathcal{D}(-2J_x^{(11)} + J_x^{(12)}) \tag{9}$$

where $J_z^{(11)} = J_z^{(22)} = 0.5J_x^{(11)} = 0.5J_x^{(22)} = \sum_{n=1}^{\infty} \frac{1}{8n^3} =$ while for dipoles of different sublattices $J_z^{(12)} = J_z^{(21)} = \sum_{n=1}^{\infty} \frac{-1+3\Lambda^2}{(n^2+\Lambda^2)^{3/2}}$, $J_x^{(12)} = J_x^{(21)} = \sum_{n=1}^{\infty} \frac{1-3n^2}{(n^2+\Lambda^2)^{3/2}}$. Solving for $\mathcal{K}$ yields

$$\mathcal{K}_c^\Lambda = \mathcal{D}\left(\frac{3}{4}\sum_{n=1}^{\infty}\frac{1}{n^3} + \sum_{u=\sqrt{1+\frac{\Lambda}{a}^2}}^{\infty}\frac{1}{u^3}\right) = 2\mathcal{D}\left(\frac{3\zeta(3)}{4} - \frac{1}{2}\psi^{(2)}\left(\sqrt{\Lambda^2+1}\right)\right) \tag{10}$$

where ψ is the PolyGamma function and ζ the Riemann zeta function.

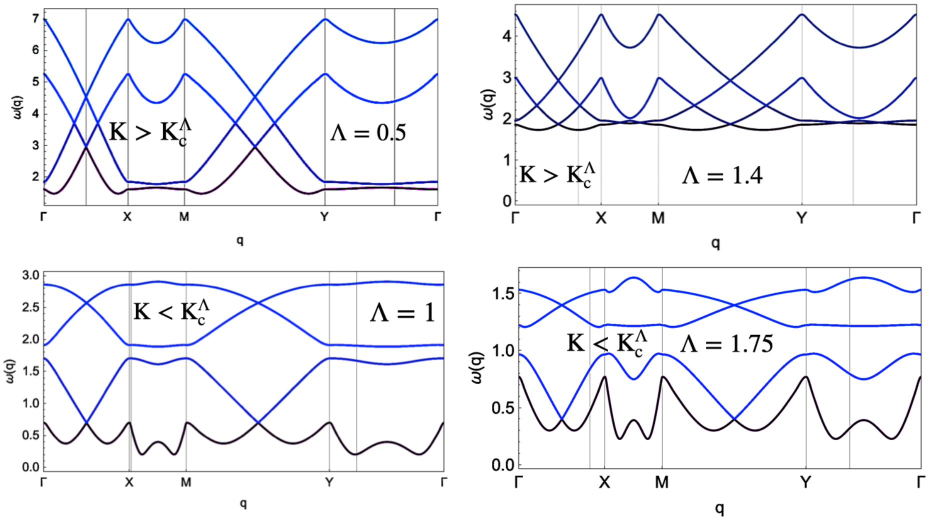

FIG. 6: Magnon spectrum of lattices with $\kappa = \pi/4$. Top panel: $K > K_c^{\Lambda}$, $\Lambda = 0.5$ (left) and $K > K_c^{\Lambda}$, $\Lambda = 1.4$ (right). Lower panel: $K < K_c^{\Lambda}$, $\Lambda = 1$ (left) and $K < K_c^{\Lambda}$, $\Lambda = 1.75$ (right).

E. Dipolar sums for transverse magnon modes

In section III, we need the dipolar sums along the $\hat{x}$ and $\hat{y}$ directions. They read respectively,

$$J_x^{(11)} = -\frac{1}{4}\sum_{n=1}^{\infty}\frac{\exp(2\imath qn)}{n^3} = -\frac{1}{4}s_1 \sim -\frac{2}{(8\cos 2q - 1)} \tag{11}$$

$$J_x^{(12)} = \sum_{n=0}^{\infty}\left(\frac{\exp((2n+1)\imath q)}{((2n+1)^2+\Lambda^2)^{3/2}} - \frac{(2n+1)^2\exp((2n+1)\imath q)}{((2n+1)^2+\Lambda^2)^{5/2}}\right) \sim \Lambda^2 s_4 \tag{12}$$

$$J_y^{(11)} = \frac{1}{8}\sum_{n=1}^{\infty}\frac{\exp(2\imath qn)}{n^3} = \frac{1}{8}s_1 \sim \frac{1}{8\cos 2q - 1} \tag{13}$$

$$J_y^{(12)} = \frac{\exp((2n+1)\imath q)}{((2n+1)^2+\Lambda^2)^{3/2}} \sim s_3 \tag{14}$$

F. Magnon spectrum of stripes for $\kappa = \pi/4$

Figure 6 and 7 compare the magnon spectrum of lattices and stripes with κ_4 in the collinear and parallel magnetic states (low versus high anisotropy).

G. Effective model.

1. *Dirac magnons in 1D*

The band touching at $q_0 = \frac{G_x}{2}$ for chains with Λ_1 can be seen as a single Dirac point around which the frequency dispersion for both bands can be approximated by a linear function. The singular structure of the frequency dispersion near the band touching can be studied using degenerate perturbation theory. For the magnon hamiltonian studied above it takes the form

$$H_p = H_0 + V_p$$

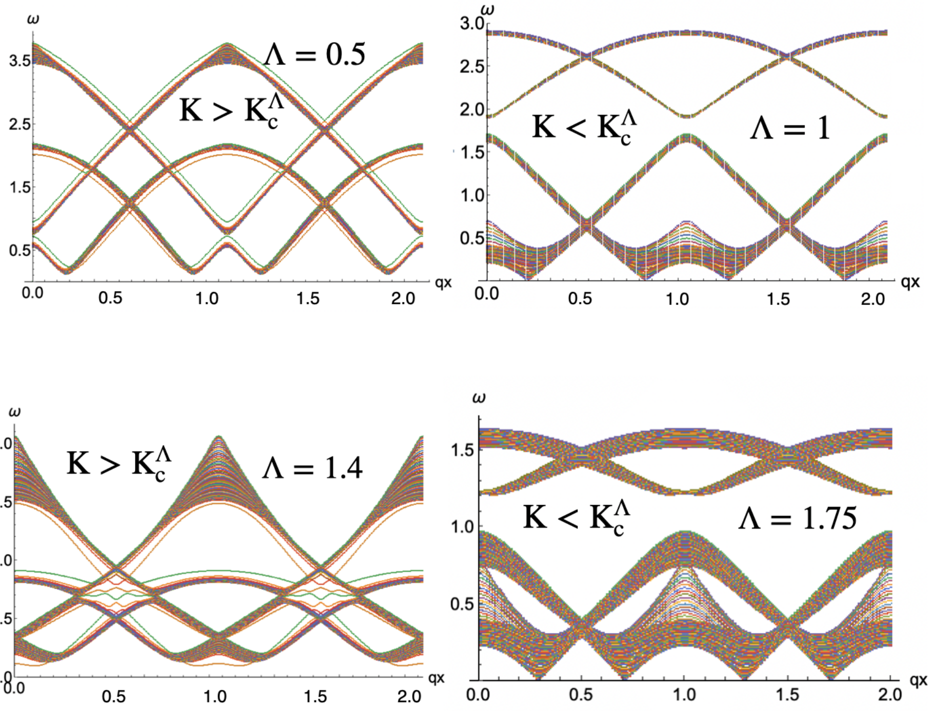

FIG. 7: Magnon spectrum of stripes with $\kappa = \pi/4$. Top panel: $K > K_c^\Lambda$, $\Lambda = 0.5$ (left) and $K < K_c^\Lambda$, $\Lambda = 1$ (right). Lower panel: $K > K_c^\Lambda$, $\Lambda = 1.4$ (left) and $K < K_c^\Lambda$, $\Lambda = 1.75$ (right).

with the band touching at $p_1 = (q_0, \Lambda_1)$ and $V_p = H_p - H_0$. At the touching point H_0 has twofold degenerate eigenstates d_j (j=1,2) with eigenfrequency $\omega_0 = 0.35$ (> 0), that satisfies $H_0 d_j = \omega_0 \sigma_z d_j$. Diagonalizing H_0 one gets

$$d_1 = (0, -\sqrt{3}/3, 0, \pi/4) \tag{15}$$

$$d_2 = i(0, -\pi/4, 0, -\sqrt{3}/3) \tag{16}$$

$$\tag{17}$$

On introducing the perturbation V_p, the degeneracy is split into two frequency levels. One gets a 2 by 2 hamiltonian h_p formed by the twofold degenerate eigenstates which in Fourier space reads,

$$h_p = \begin{pmatrix} d_1^\dagger V_p d_1 & d_1^\dagger V_p d_2 \\ d_2^\dagger V_p d_1 & d_2^\dagger V_p d_2 \end{pmatrix}$$

The matrix elements are such that $d_1^\dagger V_p d_1 = -d_2^\dagger V_p d_2$ and $d_1^\dagger V_p d_2 = d_2^\dagger V_p d_1$ Renaming $d_1^\dagger V_p d_1 = f_1(q, \Lambda)$ and $d_1^\dagger V_p d_2 = f_2(q, \Lambda)$, h_q becomes

$$h_q = \begin{pmatrix} f_1(q,\Lambda) & f_2(q,\Lambda) \\ f_2(q,\Lambda) & -f_1(q,\Lambda) \end{pmatrix} = f_1 \sigma_z + \imath f_2 \sigma_x$$

Expanding f_1 and f_2 near the point p_1 yields $f_1 \sim \beta(\Lambda - \Lambda_1)$, $f_2 \sim -\imath\nu(q - q_0)$ at linear order in q and Λ, and with $\nu > 0$ always and $\beta = 0.01$. Therefore h_q can be written like

$$h_q \sim \imath\nu(q - q_0)\sigma_x + \beta(\Lambda - \Lambda_1)\sigma_z \tag{18}$$

near the band touching point the effective hamiltonian becomes

$$\mathcal{H}_{\text{eff}} = \omega_0 \sigma_0 + \nu(q_x - q_0)\sigma_x + m\sigma_z \tag{19}$$

We identify the mass term $m = \beta(\Lambda - \Lambda_1)$ which cancels out at $\Lambda = \Lambda_1$ at the band crossing point.
